# Effect of Plasma-Activated Water Bubbles on *Fusarium graminearum*, Deoxynivalenol, and Germination of Naturally Infected Barley during Steeping

**DOI:** 10.3390/toxins15020124

**Published:** 2023-02-03

**Authors:** Ehsan Feizollahi, Urmila Basu, Rudolph Fredua-Agyeman, Brasathe Jeganathan, Lusine Tonoyan, Stephen E. Strelkov, Thava Vasanthan, Arno G. Siraki, M. S. Roopesh

**Affiliations:** 1 Department of Agricultural, Food and Nutritional Science, University of Alberta, Edmonton, AB T6G 2P5, Canada; 2 Applied Pharmaceutical Innovation, University of Alberta, Edmonton, AB T6G 2R3, Canada; 3 Faculty of Pharmacy and Pharmaceutical Sciences, College of Health Sciences, University of Alberta, Edmonton, AB T6G 2R3, Canada

**Keywords:** plasma-activated water, *Fusarium graminearum*, vomitoxin, deoxynivalenol, naturally contaminated barley, qPCR, germination, ESR

## Abstract

Contamination of barley by deoxynivalenol (DON), a mycotoxin produced by *Fusarium graminearum*, causes considerable financial loss to the grain and malting industries. In this study, two atmospheric cold plasma (ACP) reactors were used to produce plasma-activated water (PAW) bubbles. The potential of PAW bubbles for the steeping of naturally infected barley (NIB) during the malting process was investigated. The PAW bubbles produced by treating water for 30 min using a bubble spark discharge (BSD) at low temperature resulted in the greatest concentration of oxygen-nitrogen reactive species (RONS). This treatment resulted in 57.3% DON degradation compared with 36.9% in the control sample; however, the same treatment reduced germination significantly (*p* < 0.05). Direct BSD ACP treatment for 20 min at low temperature and indirect treatment for 30 min increased the percentage of germinated rootlets of the seedlings compared with the control. Considering both the DON reduction and germination improvement of barley seeds, continuous jet ACP treatment for 30 min performed better than the other treatments used in this study. At higher temperature of PAW bubbles, the concentration of RONS was significantly (*p* < 0.05) reduced. Based on quantitative polymerase chain reaction (qPCR) analysis and fungal culture tests, the PAW bubble treatment did not significantly reduce infection of NIB. Nonetheless, this study provides useful information for the malting industry for PAW treatment optimization and its use in barley steeping for DON reduction and germination improvement.

## 1. Introduction

The contamination of agricultural products with fungi and their secondary metabolites (mycotoxins) creates major challenges for the food industry. *Fusarium graminearum* is an ascomycete fungus that causes fusarium head blight in wheat and barley [1]. *F. graminearum* produces deoxynivalenol (DON), a type b trichothecene mycotoxin [2], and the consumption of foods contaminated with DON can affect animal and human health, causing nausea, vomiting, headaches, diarrhea, dizziness, fever, and other acute or chronic toxicities [3]. Atmospheric cold plasma (ACP) is one of the most explored novel antimicrobial interventions with mycotoxin degradation potential [4]. Plasma is the fourth state of matter and is comprised of UV light and reactive species including positive and negative ions, free radicals, atoms and molecules in the ground or excited states. In cold plasma, the temperature of ions and uncharged molecules is close to room temperature, whereas electrons have much higher temperatures [5]. Cold plasma can be used to treat water to generate plasma-activated water (PAW), which alters the physicochemical properties of water such as the oxidation reduction potential (ORP), pH, reactive species type, and concentration [6]. The physicochemical properties of PAW are largely affected by the plasma power supply and production system, configuration of electrodes, treatment time, type of working gas, process parameters such as voltage and frequency, gas flow rate, etc. [7].

Barley (*Hordeum vulgare*) is extensively used in the malting and brewing industries. Steeping of the barley grains in water is one of the crucial steps for making a barley malt. If *F. graminearum* is present in the grain, it can potentially grow during the steeping process and cause quality issues in barley malt. Furthermore, if DON is present in the barley grains, it may end up in the final products, if the barley malting unit operations cannot degrade this mycotoxin. The antifungal properties of PAW have been previously reported [1,8]. In a recent study by Guo, Wang, Xie, Jiang, Li, Li, Li, Liu and Lin [1], microbial activity of *F. graminearum* spores, including mycelial growth, conidial germination, and disease development, were significantly inhibited by PAW treatment of spiked wheat samples. In addition, PAW improved the germination of the seeds [9,10], which was attributed to its physicochemical properties. There are no reports, however, regarding the effect of PAW treatment on *F. graminearum* of naturally infected barley (NIB) and DON. Previous studies have focused on pure culture or artificially spiked grains, but no study has yet used naturally infected grain, which represents the samples treated at an industrial scale. Trichothecenes are produced by different genera including *Fusarium*, *Myrothecium*, *Spicellum*, *Stachybotrys*, *Cephalosporium*, *Trichoderma*, and *Trichothecium* [11]. DON as a type B trichothecene is produced by *Fusarium* spp., [2]. Biosynthesis of trichothecenes in *Fusarium* spp. begins with the cyclization of farnesyl pyrophosphate and is catalyzed by terpene cyclase trichodiene synthase (Tri5) that is encoded by the (*Tri5*) gene [11]. The *Tri6* gene is one of the genes regulating the expression of other *Tri* genes for the biosynthesis of trichothecenes [12]. *EF1A* is involved in the protein translation machinery of eukaryotic cells and has been shown to be a powerful tool for distinguishing related *Fusarium* species [13]. Actin is the most abundant protein in most eukaryotic cells [14] and is encoded by the housekeeping actin gene that can be used for *F. graminearum* identification. The genes can be used for *F. graminearum* detection and relative quantification based on quantitative polymerase chain reaction (qPCR).

In this study, we assessed the potential for the use of PAW in the steeping of barley for DON degradation, *F. graminearum* inactivation, and improving the germination of NIB grain. We investigated the characteristics of PAW bubbles produced using a bubble-spark discharge (BSD) reactor and a continuous jet (CJ) ACP. We also fabricated a process unit with a Venturi tube and plasma jet for continuously producing PAW bubbles for improving the diffusion of plasma-reactive species into water. The PAW bubbles produced by these units were characterized by measuring the pH, ORP, and reactive species concentration. Then, the effects of PAW bubbles on DON degradation and germination improvement of NIB were measured. Finally, the antifungal effect of PAW bubbles on NIB was assessed by the use of fungal culture and qPCR techniques.

## 2. Results and Discussion

### 2.1. Characteristics of PAW Bubbles

The physicochemical properties of PAW bubbles were affected due to the formation of RONS (Table 1). The reactions for the formation of hydrogen peroxide (H_2_O_2_), nitrate (NO_3_^−^), nitrite (NO_2_^−^), and ozone (O_3_) were reported in our previous manuscript [15]. As the ACP treatment time increased, we observed a significant (*p* < 0.05) increase in the ORP and a significant (*p* < 0.05) decrease in the pH (increase in H^+^ ion concentration) of the treated water (Table 1). The greatest ORP was obtained in PAW bubbles produced using 30 min BSD treatment (treatment B), which had a good correlation with the concentration of other reactive species, i.e., H_2_O_2_, NO_3_^−^, NO_2_^−^, and O_3_. In addition, DW had the least ORP and the highest pH. Also, treatment D had higher pH and lower ORP compared to other treatments which were due to weaker plasma in treatment D and the higher amount of water used in this treatment. The increase in ORP was likely due to an increase in the concentration of active ions and oxidizing species such as H_2_O_2_, HNO_3_, HNO_2_^−^, and O_3_ in PAW bubbles [7]. It should be noted that pH affects the ORP; in acidic pH, hydrogen ions (H^+^) increase the ORP, whereas in alkaline pH, hydroxide ions (OH^−^) decrease the ORP value [8].

Since PAW was used to steep the grains for 5 h, we also measured the RONS concentration after 5 h storage of PAW to determine its physicochemical properties. For the PAW bubbles produced by 20 min BSD treatment (treatment A), the concentrations of all the RONS decreased significantly (*p* < 0.05) after 5 h storage, except for NO_3_^−^. After 5 h, the concentrations of all the RONS in the PAW bubbles produced by treatment B decreased significantly (*p* < 0.05) (Table 1). In the case of treatment D (PAW bubbles produced by 30 min CJ ACP), the % reduction in the concentrations of H_2_O_2_ (6.67%) was smaller after 5 h storage compared to those for treatments A (37.3%) and B (55.8%) after storage. However, higher % reduction in the concentration of O_3_ (62.5%) was observed in PAW bubbles produced by treatment D after 5 h storage compared to treatments A (60.6%) and B (58.8%) after 5 h storage. Treatment D (CJ ACP) produced considerably fewer concentrations of RONS in the PAW bubbles compared to BSD ACP treatment. It is possible to have low reaction rates of RONS with each other, as their concentration was too low in the PAW bubbles produced by CJ ACP. However, this assumption may not be accurate as the highest ozone reduction during storage happened for PAW bubbles produced by CJ ACP. During treatment D, bubble formation happens in the Venturi, and hence RONS could be captured inside the bubbles. The reduction in O_3_ concentration during storage in the PAW bubbles could be due to the reaction of O_3_ with H_2_O_2_, and NO_2_^−^ (Equations (1) and (2)). H_2_O_2_ was formed mainly from water decomposition and subsequent recombination of ^•^OH radicals (reaction 7) [16]. Due to reactions 1 and 3, the concentration of H_2_O_2_ was also reduced during storage [7]. Reactions 2, and 3 could also be the reason for the decrease in the concentration of NO_2_^−^ during storage. Moreover, NO_3_^−^ had the greatest concentration in PAW compared with other RONS due to its formation via reactions 2 and 3 in addition to formation via ACP. It is also possible that NO_2_ and NO, which are formed via ACP, react with water and form stable RONS (NO_3_^−^, NO_2_^−^) via reactions 4–5. The ozone concentration was very high in the PAW bubbles treated with BSD (treatments A, B). The stability of ozone was the greatest at pH 2–6 [17], and this could be the reason for having a large concentration of ozone even after 5 h of storage.

The ORP and pH of the water remained stable after 5 h of storage (treatments A, B, D), suggesting the presence of oxidizing and acidic agents in the solution. Nitrous and nitric acid, as well as H_2_O_2_, caused the PAW acidification, in which nitrous and nitric acid were formed from nitrate and nitrite ions in the solution following reactions 4–5 [6,18]. Julák, Hujacová, Scholtz, Khun and Holada [16] reported that HNO_2_ had a negligible effect on the acidity of the PAW and the overall acidity was assigned to HNO_3_. This was because HNO_2_ could be decomposed following reaction 6 within a few minutes [16]. With the reaction of H_2_O_2_ with water, acidic H_3_O^+^ ions would be formed and this could also decrease the pH [19]. Wu, Liu, Chiang, Lin, Lin, Chang and Wu [8] used oxygen as the carrier gas for jet plasma to produce PAW and the pH of the PAW was not significantly different from the untreated water. This suggests the importance of the presence of nitrogen atoms in the carrier gas for acidifying the PAW via HNO_3_ generation. Similar to our study, the pH of PAW did not change in the previous studies, even after one year of storage at 4 °C [16].

The PAW bubbles produced by treatment B were formed at room temperature (~23 °C), and by surrounding the treatment container with ice cubes. During treatment, the temperature of PAW bubbles increased from room temperature to 51.9 ± 1.2 °C after treatment B. However, the final temperature was 12.3 ± 0.6 °C, when the ice cubes were placed around the treatment container. The results show that at high water temperature, the concentrations of reactive species were considerably lower. This could be due to the higher reaction rates at higher temperatures, which resulted in faster decomposition of the major reactive species (H_2_O_2_, NO_3_^−^, NO_2_^−^, and O_3_) in PAW bubbles.

The characteristics of PAW bubbles depend upon several factors, including the ACP generation method, quantity of water, working gas, operating parameters of the ACP, and treatment time. As the plasma generated by CJ was considerably weaker than BSD, and due to the larger quantity of water used, lower concentrations of RONS in PAW bubbles prepared by CJ ACP were observed.

(1) $$O_{3}\left(\mathrm{aq}.\right)+H_{2}O_{2}\to {\mathrm{HO}}^{•}{+\mathrm{HO}}_{2}^{•}{+O}_{2}$$

(2) $${\mathrm{NO}}_{2}^{-}+O_{3}\to {\mathrm{NO}}_{3}^{-}{+O}_{2}$$

(3) $${\mathrm{NO}}_{2}^{-}+H_{2}O_{2}\to {\mathrm{NO}}_{3}^{-}{+H}_{2}O$$

(4) $${2\mathrm{NO}}_{2}+H_{2}O\to {\mathrm{NO}}_{2}^{-}{+\mathrm{NO}}_{3}^{-}{+2H}^{+}$$

(5) $$\mathrm{NO}+{\mathrm{NO}}_{2}{+H}_{2}O\to {2\mathrm{NO}}_{2}^{-}{+2H}^{+}$$

(6) $${\mathrm{HNO}}_{2}\to {\mathrm{NO}+\mathrm{NO}}_{2}{+H}_{2}O$$

(7) $${\mathrm{HO}}^{•}{+\mathrm{HO}}^{•}\to H_{2}O_{2}$$

### 2.2. EPR Spectroscopy

**Figure 1:**
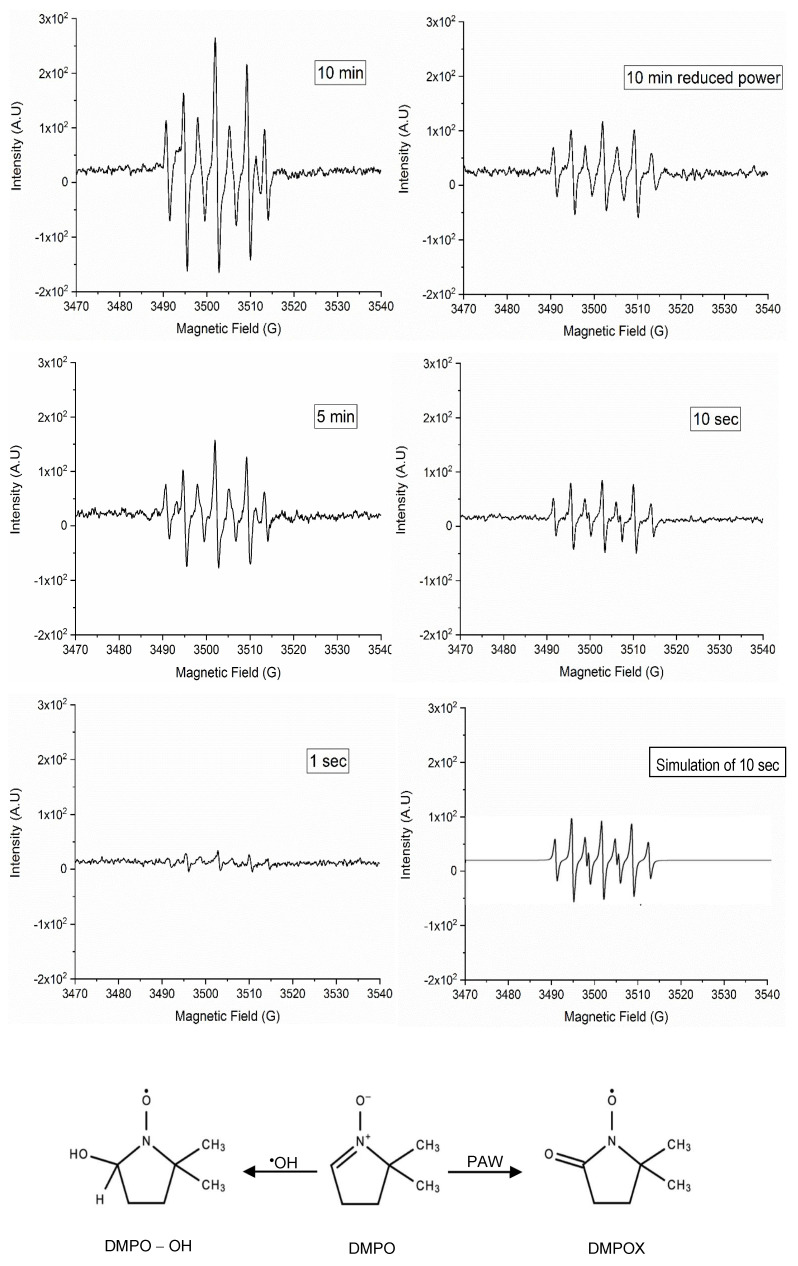
Electron paramagnetic resonance spectra of 5 mL deionized water+50 mM DMPO treated with BSD ACP at different times and power, and the chemical structure of DMPO, DMPO−OH, and DMPOX (adapted with permission from Chen, Li, Chen and Kong [21]. 2023. AIP Publishing). The simulated spectrum had hyperfine splitting constants of a^N^ = 7.26 G, a^H(2)^ = 4.02 G, and a correlation parameter of r = 0.98. The EPR spectrum was simulated using WinSim2002 (NIEHS/NIH).

Hydroxyl radical (^•^OH) is one of the most important reactive oxygen species that can be formed in PAW through reaction of O_3_ with H_2_O_2_ (reaction 1) and through the dissociation of water molecules during ACP treatment [6]. DMPO was used to spin trap the ^•^OH. The reaction of DMPO with short-lived ^•^OH formed a long-lived spin adduct, DMPO-OH (2-hydroxy-5,5-dimethyl-1-pyrrolidinyloxy) (Figure 1), which had a unique ESR spectrum with a peak intensity ratio of 1:2:2:1 [20]. However, the EPR results showed a dominant 7-peak spectrum rather than the characteristic 4-peak spectrum of DMPO-OH. This spectrum was ascribed to DMPOX (5,5-dimethyl- 2-pyrrolidone-N-oxyl) based on the literature [21,22]. Similar results were reported by Liu, Liu, Chen, Yang, Li, Rong, Chen and Kong [20], Chen, Li, Chen and Kong [21] and Gao, et al. [23], where DMPO was oxidized to DMPOX after plasma treatment. We observed that the species formed initially regardless of the conditions, and we simulated the spectrum (Figure 1, Simulation of 10 sec) based on the 10 sec PAW treatment, which is similar to the previous report on the simulation of this species (Barr and Mason, 1995. JBC. 270:12709-12716). Conditions that exceeded the 10 sec PAW treatment continued to produce a dominant DMPOX spectrum, but another DMPO oxidation product most likely related to DMPOX was observed with 5 and 10 min treatments. As oxidation occurred at 10 min treatment time, the input voltage was reduced from 150 V to 100 V in which the weakest plasma was generated at this voltage and no plasma was generated at voltages below 100 V. The spectrum indicated that the DMPO was oxidized again; however, the intensity of the peaks was lower. Furthermore, the results showed that increasing the treatment time allowed the spin adducts to accumulate and intensified the peaks’ height. As longer treatment times oxidized the DMPO, the BSD ACP treatment was applied to the water for one second. Interestingly, the DMPO was oxidized to DMPOX again, which indicated the presence of oxidizing agents even after a very short ACP treatment. We also added 5% dimethyl sulfoxide (DMSO), a potent ^•^OH scavenger, however, DMPO was oxidized, and an asymmetric seven-peak spectrum was observed (not shown in Figure 1). This demonstrated that the oxidation of DMPO was not due to ^•^OH. In a previous study, sodium azide (scavenger of ^1^O_2_) was used along with DMPO. The presence of 10 mM of sodium azide resulted in a complete reduction of DMPOX concentration, which suggested the possibility of oxidation of DMPO via ^1^O_2_ [21]. Overall, the spectrum of DMPO-OH was not observed, as the potential of DMPO oxidation was greater than its potential for trapping the ^•^OH radicals in PAW.

### 2.3. Efficacy of PAW Bubble Treatments on DON Reduction

As shown in Table 2, different PAW bubble treatments did not have a significant (*p* > 0.05) impact on DON reduction compared with the control sample. This was due to a high standard deviation in the naturally contaminated samples. However, comparison of the minimum and maximum amount of DON within the treated samples shows the impact of the PAW bubble treatments on DON degradation. Among the different treatments, direct PAW production for 30 min using BSD at low temperature (surrounded by ice) (treatment B) resulted in the highest reduction of DON compared with the other treatments. Treating NIB grains by treatment B (direct treatment) resulted in higher DON degradation, with 57.3% reduction relative to treatment C (indirect treatment) with 31.2% reduction. Treatment B also had the highest concentration of long-lived RONS, i.e., H_2_O_2_, NO_3_^−^, NO_2_^−^, and O_3_. Among these, H_2_O_2_ and O_3_ could play a role in DON degradation; however, NO_3_^−^ and NO_2_^−^ have no significant (*p* > 0.05) impact on DON degradation [24]. In the direct treatment, there are short-lived free radicals such as (^•^OH), superoxide (O_2_^−^), singlet oxygen, and high energy electrons aside from the RONS, which could be the main reason for the greater degradation of DON in the direct vs. indirect treatment. Singlet oxygen is very unstable and reacts with electron-rich double bonds. DON has an olefinic double bond at the C9–C10 position, which is one of the most reactive sites for O_3_ and singlet oxygen [25]. The results from treatments A and B demonstrate the higher efficacy of the longer treatment time. Higher RONS concentration after treatment B might play a role in the higher DON degradation efficacy. In addition, under longer treatment times, a larger amount of short-lived reactive species could react with the grain surface and DON molecules, resulting in higher DON degradation efficacy. Another factor is the pH of the solution, which may influence the degradation of DON. The degradation of DON was reported as 30 and 60% at pH 3 and 2, respectively [26]. However, in our experiments, the pH of treatments A and B were similar, but the DON degradation rates were different. Hence, factors other than pH play a more important role in DON degradation in naturally infected grain.

During the barley steeping process, DON can be possibly washed away, resulting in the reduction of DON even in the control NIB samples (Table 2). After steeping the barley in PAW bubbles produced directly by CJ reactor for 30 min (treatment D), there was a 7% increase in DON reduction compared with the control (Table 2). However, 1 h of direct PAW bubble (treatment E) steeping treatment did not increase the DON reduction. This could be due to the larger quantity of water used and the lower concentrations of RONS (Table 1), which seem insufficient for the DON degradation.

In a previous study on using PAW produced by plasma jet at 4.4 kV for DON degradation, there was no significant (*p* > 0.05) reduction in DON in the PAW-treated barley samples [27]. Lower degradation of mycotoxins in artificially infected grains compared with the pure form of the mycotoxin was reported in previous studies [28,29]. Severely *Fusarium*-infected grains have deeper infections [30] and DON can be present in the inner layers of the grain. This could be the main reason for the lower efficiency of PAW bubbles in NIB grains compared with artificially infected grains, as RONS and other active species in plasma cannot penetrate the deeper layers of the grains.

### 2.4. Germination of NIB Grains after PAW Bubble Treatments

To determine the potential utility of the PAW bubble treatment in the steeping process of barley malting, the germination of the NIB grains was assessed. The greatest germination of the barley rootlets was after 30 min indirect PAW bubble treatment (treatment C) using the BSD reactor (Table 3). The direct 30 min BSD treatment at low temperature (surrounded by ice) (treatment B) had a significantly (*p* < 0.05) negative impact on the germination of the rootlets and acrospires. The direct PAW bubble treatment using BSD (treatments A, B) negatively affected the acrospire germination; however, rootlet germination could be negatively/positively affected based on the treatment time (i.e., long-lived, and short-lived RONS concentration). Comparing treatment B with treatment C demonstrates the importance of the short-lived RONS and free radicals on the germination efficacy of the ACP system in the direct treatment, as in treatment B there is a constant production of the short-lived RONS and free radicals, which negatively affected the germination of the grain. However, in the 20 min direct PAW bubble treatment using the BSD reactor (surrounded by ice) (treatment A), the germination of the rootlets was slightly reduced whereas the acrospire germination was significantly (*p* < 0.05) declined compared with treatment C. The results shows that the long-lived RONS had improved the germination parameters, whereas short-lived RONS, or free radicals, decreased the germination of the NIB seeds.

Similarly, in a previous study by Chen, Chen, Cheng, Peng, Liu, Ma, Liu and Ruan [27], the germination of barley grains decreased with increasing the PAW treatment time. It should be noted that the state of the seeds used in different studies is important to consider when comparing the results of these studies, as the efficacy of PAW is different for fresh mature seeds compared with ripened seeds [9]. The 30 min direct PAW bubble treatment using the CJ reactor (treatment D) increased the germinated rootlets by 5% however, it was not significant (*p* > 0.05) compared with its respective control. This is because the concentration of RONS was insufficient to cause any change in the germination of the grain.

In response to environmental cues, seeds produce H_2_O_2_, NO_3_^−^, ^•^OH, and NO to release physiological dormancy by activating molecular signaling pathways [9]. It was demonstrated that PAW could trigger many of these pathways, such as abscisic acid degradation and gibberellin synthesis and signaling, which might result in dormancy release of the seed [9]. In a study by Than, et al. [31], the optimum germination of lettuce seeds was obtained after 15 min PAW treatment, whereas longer treatment times resulted in lower germination rates. In another study, longer treatment times of the barley seeds using microwave discharge resulted in lower shoot and root lengths in the seedlings. It was determined that using the gas mixtures with high NO content in a microwave discharge treatment inhibited barley germination [32].

Plants produce different ROS such as H_2_O_2_, superoxide (O_2_^•−^), ^•^OH, and singlet oxygen (^1^O^•^_2_) in response to environmental stresses. Overproduction of these ROS can cause oxidative damage to plant macromolecules and cell structures, which will lead to inhibition of plant growth and development or death [33]. In contrast, at moderate amounts, ROS, like H_2_O_2_, may be beneficial for plant germination [34]. In a study by Jirešová, Scholtz, Julák and Šerá [10], the effect of PAW on wheat germination was compared with artificial PAW prepared from H_2_O_2_ and nitric acid. There were no significant (*p* >0.05) differences between the efficacy of both the PAW treatments on percentage germination, root and shoot length, or fresh/dry weights of the roots and shoots. This suggested that the major RONS of PAW involved in the germination are H_2_O_2_, NO_3_^−^, and NO_2_^−^_._ Moreover, the RONS generated by ACP can impact the expression of germination-related genes, the activity of growth hormones, antioxidants and enzymes, changes in sugar, chlorophyll, amino acids, and water absorption [35].

The activity of the α-amylase, β-amylase, and β-glucanase enzymes was also assessed, as these enzymes are involved in the germination of barley grain. The percentage germination of rootlets and acrospire in Control D and treatment C samples were not significantly different from treatment D, hence their enzymatic activity was not measured. The activity of α-amylase, and β-glucanase increased after malting of the NIB grains. There were no significant (*p* > 0.05) differences in α-amylase activity between the DW and PAW bubble-treated samples. Treatment A had the highest α- and β-amylase activities, which correlated well with its rootlet germination rate. The β-glucanase increased significantly (*p* < 0.05) in all of the PAW bubble-treated samples, except for treatment B, which also had the lowest germination based on the rootlets and acrospire germination. The results from the enzymatic activity measurement suggests that in longer ACP treatment time, β-glucanase activity is negatively affected, which influenced the germination of the grain. As discussed in this section, there are numerous parameters involved in the germination of a seed and RONS from plasma can differently impact any of these, thereby changing the percentage germination. More study is needed in this regard to comprehend fully the mechanism(s) of long-lived and short-lived reactive species on regulating the germination of a plant.

### 2.5. Effect of PAW Bubble Treatments on Microflora and F. graminearum in NIB Grains

**Figure 2:**
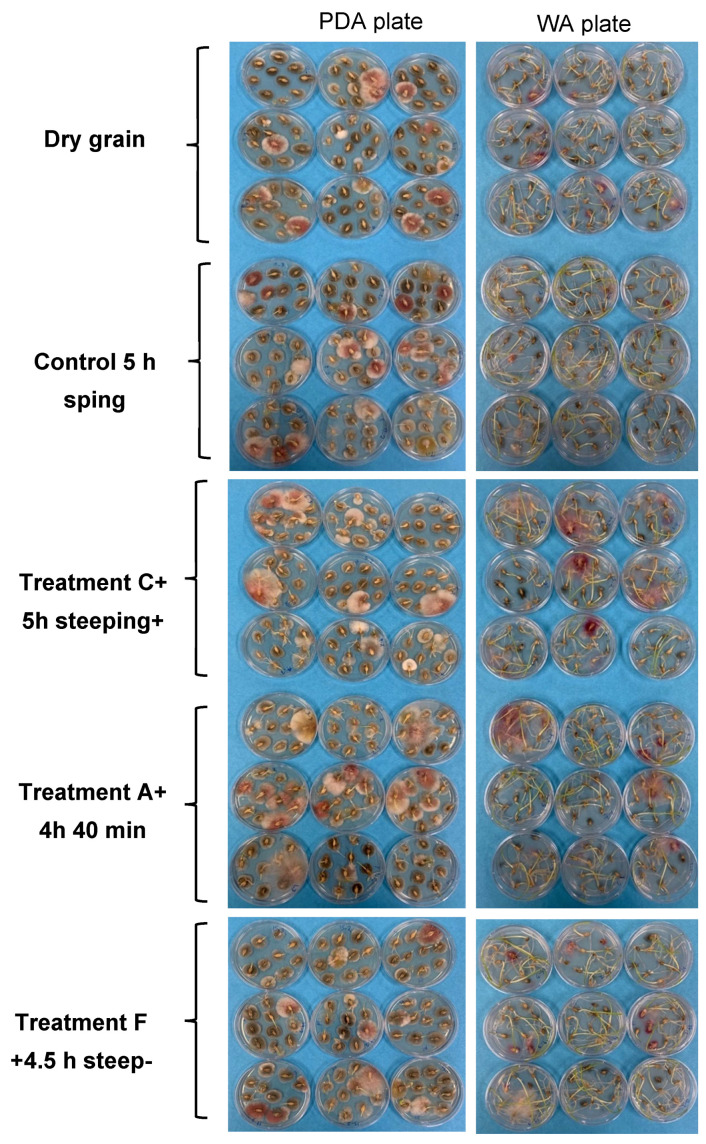
Effect of PAW bubble treatments on natural microflora, *F. graminearum* and germination of naturally infected barley grains. Treatment A: Direct 20 min BSD (ice surrounding); Treatment C: Indirect 30 min BSD; Treatment F: Direct 30 min batch-jet (BJ). Seeds were plated on potato dextrose agar medium (PDA) and water agar (WA).

To determine the antimicrobial activity of PAW bubbles, the treated and untreated NIB grains were plated on PDA agar, which confirmed the presence of other microflora in addition to *F. graminearum* (Figure 2). The average diameter of microbial colonies recovered from NIB following the various treatments is summarized in Table 4. Although the PAW bubble treatments did not have a significant (*p* >0.05) effect on colony diameter (Figure 2), there was a reduction in the colony diameter of the steeped vs. dry grain (grain without steeping in water) samples. There was no significant (*p* > 0.05) difference in the proportion of *F. graminearum*-infected grains, as assessed on PDA, between the treated and untreated samples. Similarly, the colonies of *F. graminearum* (confirmed by its morphology under microscope) recovered seemed to be of similar size based on visual inspection of the treated and untreated samples. Nonetheless, the sample size used in this work was small, and further testing may be required to confirm the absence of an effect of PAW bubble treatment on the microbiota associated with NIB.

The NIB grains were also incubated on WA plates to assess their germination. As expected, dry grain had significantly (*p* < 0.05) lower shoot length compared with other samples. However, percentage germination and shoot length were not significantly (*p* > 0.05) different between the treated and control grains. This was probably due to favorable conditions on the WA plates such as the presence of water and nutrients for germination of the grains, which overcame any differences in the relative viability of the seeds. In addition, as was observed on the PDA plates, the number of *F. graminearum*-infected grains was not significantly (*p* > 0.05) different between the treated and untreated samples incubated on the WA medium (Table 4, Figure 2). In a previous study, the growth of *F. graminearum* was significantly inhibited following PAW treatment of *F. graminearum* in culture [1]. Moreover, the growth rate of *F. graminearum* was significantly (*p* < 0.05) reduced after treating the fungus with gliding arc plasma [36]. There are other studies on the reduction of the growth of *Aspergillus parasiticus* and *Aspergillus flavus* on inoculated groundnuts [37], *Penicillium italicum* on inoculated kumquat [38], *A. parasiticus* and *A. flavus* on inoculated hazelnuts [39] after ACP/PAW treatment. These studies assessed the effect of ACP/PAW treatment in fungal cultures or grains artificially inoculated with the fungi. In our study, naturally infected barley grains were used, in which *F. graminearum* and other microflora could reside in the inner layers of the grain, especially in severely infected grains. This would reduce the capacity of RONS from PAW to react with the fungi and could be the main reason for the apparent inefficacy of the PAW treatment on reducing the infection of NIB grains.

In a study by Xu, et al. [40], 5 and 10 min PAW treatments of naturally contaminated button mushrooms reduced fungal content by approximately 1 log value. However, a 15 min PAW treatment did not affect fungal content significantly. In another study, 10 min air plasma treatment resulted in a 4% decrease in *Fusarium* in naturally contaminated field pea, and a 15 min air plasma treatment resulted in a 9% decrease in *Fusarium* in naturally contaminated blue lupin seeds [41]. Similar to our study, the efficacy of cold plasma on naturally infected grains seems to be considerably lower relative to inoculated seeds. Nonetheless, more studies are required to improve understanding of the effect of PAW bubble treatments and RONS on microflora and *F. graminearum* on or in NIB grains.

The RONS and radicals from PAW can react with the cell walls of fungi and result in the oxidation of the cell wall components such as glucose, (N-acetyl)-glucosamine, glycoproteins and glucan, and peroxidation of the lipids in the plasma membrane. This will result in the damage and perforation of the cell wall and membrane, and subsequent exposure of the intracellular components to RONS [1]. Different parameters in PAW, such as type and concentration of RONS and pH of PAW, can influence the growth of microflora. In previous studies, *F. graminearum* germination and mycelium growth were higher at pH 4 vs. pH 8 [42], and the mycelial growth of *F. graminearum* at different pH was pH 5 > 6 > 7 > 8 [43]. This shows that the pH of PAW produced from batch-jet (BJ) the ACP treatment (treatment F) (pH = 3.86) may favor the growth of *F. graminearum*. However, a previous study on *Colletotrichum gloeosporioides* inactivation by PAW determined that long-lived RONS (ozone, nitrate, and nitrite) made the greatest contributions to fungal growth inhibition, whereas acidic pH had a minor effect on fungal inactivation [8]. In addition, NO_2_ showed higher antifungal activity relative to NO_3_ [8]. The fungal biomass of *F. graminearum* is positively correlated with DON content on grains [44]. The factors that promote fungal growth, including environmental factors, growth stage, chemotype of pathogen isolate, lodging, tillage, cultivar resistance, and fungicide application [45], can affect DON accumulation on grains. Hence controlling and optimizing the aforementioned factors could be beneficial for *F. graminearum* reduction as PAW treatment did not exhibit antifungal properties.

### 2.6. Effect of PAW Bubble Treatment on Fungal Biomass of F. graminearum

*Tri5*, *Tri6*, *EF1-A* and *β-Actin* genes were used for *F. graminearum* detection and relative quantification based on qPCR [46,47,48,49], in order to confirm the results obtained with the plating technique, which indicated that the PAW bubble treatment did not have any effect on *F. graminearum* infection or growth. DON biosynthesis is governed by fifteen genes that are present over three chromosomes [50] and an acidic pH of environment encourages DON biosynthesis. Also, DON biosynthesis is induced in response to plants’ defense mechanism to fungi infection [51].

**Figure 3:**
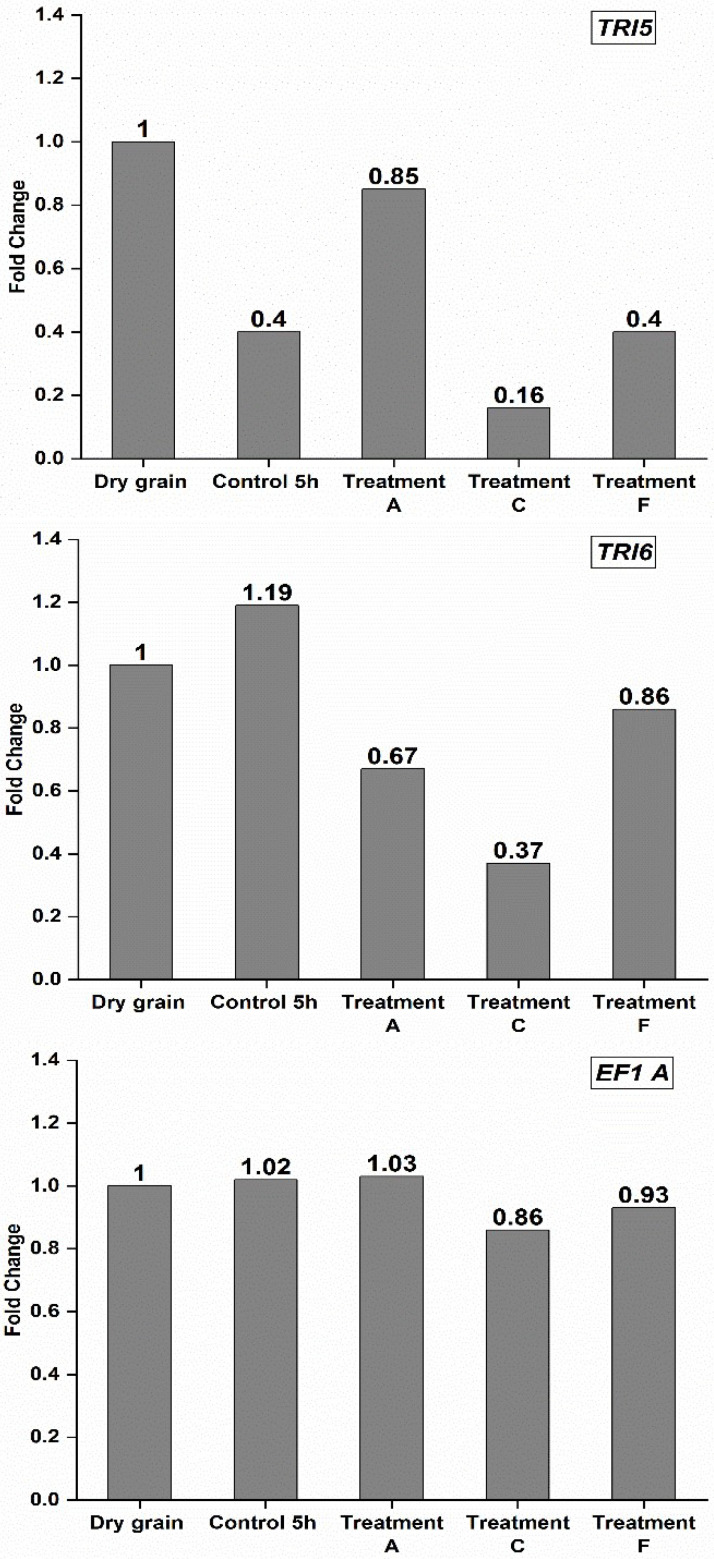
Effect of PAW bubble treatments on the relative abundance of three target genes (*Tri5*, *Tri6* and *EF1-A*) of *F. graminearum* with *β-Actin* as an endogenous reference gene, based on quantitative PCR analysis. Values are reported based on fold-change values. The fold-change was calculated using fold-change = 2^−ΔΔCt^ where, ΔCt = Ct (target gene)—Ct (reference gene), and ΔΔCt = ΔCt (treatment)—ΔCt (control). Treatment A: Direct 20 min BSD (ice surrounding); Treatment C: Indirect 30 min BSD; Treatment F: Direct 30 min BJ. Control and treatments A, C, and F were followed by steeping and air rest step before qPCR test.

RONS from ACP can damage the structure of the fungal cell walls and reach intracellular components, such as nucleic acids, and degrade them [52]. The fold-changes in relative abundance of the three target genes *Tri5*, *Tri6* and *EF1-A* (with *β-Actin* used as an endogenous control) following PAW bubble treatments are presented in Figure 3. Relative to the dry grain sample, the abundance of *Tri5* and *Tri6* was reduced in all treatments, with the strongest reduction observed in treatment C (Figure 3). In contrast, the abundance of *EF1-A* was not altered relative to the dry grain sample. There was a large standard deviation in the Ct values of the samples for each gene, likely because some grains were highly contaminated, and others were not infected. The presence of a few contaminated or infected seeds in one sample relative to others can result in a large standard deviation. This variability, combined with an apparent lack of reduction of *EF1-A*, suggests that reductions in fungal biomass because of the PAW treatments were not consistent or particularly pronounced.

## 3. Conclusions

Contamination of barley grains by mycotoxins and pathogenic fungi is one of the major issues facing the malting industry. Deoxynivalenol (DON) and *F. graminearum* are the main mycotoxin and pathogenic fungus, respectively, affecting barley seed. In this study, the potential of plasma-activated water (PAW) bubbles produced from different atmospheric cold plasma (ACP) units was assessed for DON degradation and *F. graminearum* inactivation during the steeping of barley grains. There was a positive correlation between the concentration of RONS in PAW and DON reduction. Further research is required for determining the main reactive species responsible for DON degradation in PAW. Previous research determined the efficacy of ACP/PAW treatment on inactivation of fungi and DON spiked on grains [37,38,39]. In this study we used naturally infected barley (NIB). The PAW bubble treatments used in this study were not able to inactivate *F. graminearum* on NIB grains probably due to the presence of fungi in the inner layers of the NIB grains and/or growth promotion of fungi in acidic pH. As using ACP in a gaseous state has etching effect and pH does not play a role in promoting the fungal growth, its efficacy against *F. graminearum* on naturally contaminated grains should be investigated. Our results determined that to reduce both the DON concentration and improve germination, PAW bubbles produced by the continuous ACP jet treatment (treatment D) performed better compared to the other tested ACP treatments. Collectively, the use of an optimized PAW bubble treatment can improve germination and reduce DON content in barley grains.

## 4. Materials and Methods

### 4.1. Barley Grains

Naturally infected barley grains (2-row malting variety, ‘CDC Copeland’) with high concentrations of DON (4.6–5.8 ppm) were procured from Agriculture and Agri-Food Canada, Brandon Research and Development Centre, Brandon, Manitoba. The grains were kept in Ziploc bags and stored at 4 °C until used. The initial moisture content of the grains before use was 8.6 ± 0.2% (wet basis).

### 4.2. PAW Bubble Production

**Figure 4:**
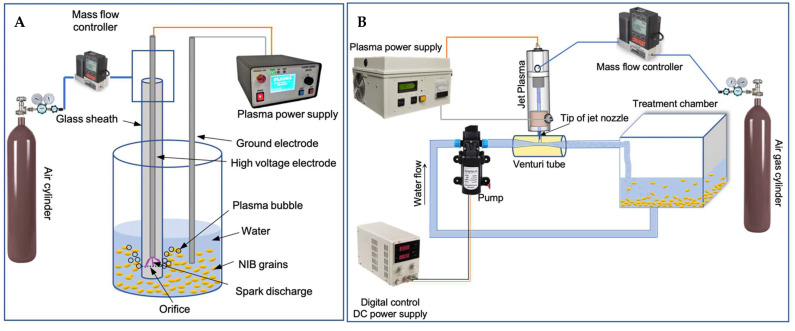
Schematic diagram of plasma activated water (PAW) bubble treatment of naturally infected barley (NIB) grains using: (**A**), bubble spark discharge atmospheric cold plasma (ACP); and (**B**), continuous jet ACP.

PAW bubbles were produced by three methods. First, a bubble spark discharge (BSD) reactor (Figure 4A) was connected to a high voltage micropulse generator Leap100 (PlasmaLeap Technologies, Sydney, Australia), with a voltage pulse of 150 V, a repetitive pulse frequency of 1000 Hz, and a duty cycle of 66 μs. The BSD reactor consisted of a stainless-steel high voltage electrode rod (4 mm outside diameter (OD)) inserted coaxially along the length of a 175-mm-length quartz tube (10 mm OD, and 1.5 mm wall thickness) with one end sealed. At 5 mm above the sealed end, eight holes were positioned radially to allow the formation of bubbles in water. The generated bubbles with certain flow rate created agitation and distribution of plasma species into the water. A Polytetrafluoroethylene tee fitting was connected to the open end of the quartz tube to supply the airflow into the tubes and to support the electrode in its place. Another stainless-steel electrode rod was used as the ground electrode during the treatments. Air was pumped at a flow rate of 1 standard liter per minute (SLPM) via a mass flow controller (MC-Series, Alicat scientific, Tucson, AZ, USA) to the reactor and 80 mL water was used, producing PAW bubbles for all the experiments using the BSD reactor. Here, plasma was generated continuously for selected times and carried by the underwater bubbles. The agitation and movement of bubbles containing the plasma species allowed further interaction of these species with water due to the large water surface areas. In the 20 (Treatment A) and 30 min (Treatment B) direct BSD treatments, ice cubes were placed around the treatment beaker to prevent a temperature increase during PAW bubble generation.

In another set of experiments, a continuous jet (CJ) ACP unit (Figure 4B) consisting of a Venturi tube mounted on the tip of the jet system’s nozzle, a water pump (Micro Diaphragm Pump, Riuty, China) and a quartz chamber (9 × 9 × 9 cm) was used for PAW bubbles production. The pump was connected to a digital control DC power supply (kd3005d, Korad technology, Dongguan, China), providing 1.8 V with 0.55–0.66 Hz frequency to the pump. The jet system was connected to a power supply (PG 100-D, Advanced Plasma Solutions, Malvern, PA, USA), providing a frequency of 3500 Hz, 70% duty cycle, output voltage of 0–34 kV, power of 300 W, and a 10 μs pulse width. Air was pumped at a flow rate of 0.5 SLPM and 180 mL water was used for all the experiments using CJ ACP. Plasma was injected into the Venturi tube using the plasma jet, with water passing through the Venturi, producing plasma-activated water bubbles. The interaction of plasma-reactive species from plasma jet with water at the Venturi created bubbles with plasma-reactive species and they were carried to the treatment chamber. Also, pumping of water through the Venturi resulted in hydrodynamic cavitation and bubble formation, leading to better interaction of plasma reactive species with water. Both the BSD and CJ ACP designs supported continuous treatment of grains with plasma-activated water bubbles and the scaling of the plasma reactor for industrial applications.

For the next set of experiments, the above jet unit was used for batch treatments. The BJ ACP was used to produce PAW bubbles and assess its effect on the pathogens and *F. graminearum* inactivation. The PAW bubbles from the BJ ACP unit could be more effective than CJ ACP due to a greater concentration of RONS in a smaller amount of PAW. Water (30 mL) was treated using the BJ ACP unit inside a beaker and the treatment parameters were similar to the CJ ACP treatment. The characteristics of the PAW bubbles produced using the BJ ACP unit was reported in our previous work.

In treatment C (indirect 30 min BSD), PAW bubbles were generated by ACP and then added to the NIB grains for steeping. However, in the direct treatments, i.e., treatment A (direct 20 min BSD surrounded by ice), treatment B (direct 30 min BSD surrounded by ice), treatment D (30 min direct CJ), and treatment E (1 h direct CJ), NIB grains were in contact with PAW inside the container during PAW generation. The various treatments are summarized in Table 5.

### 4.3. PAW Bubble Characterisation

PAW bubbles were produced by treating 80 mL water using the BSD ACP, 180 mL water using the CJ ACP, and 30 mL water using the BJ ACP units for different treatment times. The characteristics of PAW were measured indirectly after ACP treatments, where the PAW was generated first and then used for measuring the physicochemical properties of PAW. The pH and ORP values were determined using a pH meter (Fisher Scientific, Accumet AE150, Singapore) and an ORP meter (Ohaus, ST20R, Parsippany, NJ, USA), respectively. CHEMetrics kits (Midland, VA, USA) were used to determine hydrogen peroxide (K-5543) based on the ferric thiocyanate method; nitrate (K-6933) based on the cadmium reduction method; nitrite (K-7003) based on the azo dye formation method; and ozone (K-7423) based on the DPD (N,N-diethyl-p-phenylenediamine) oxidation method in PAW bubbles. The color change in the kits was measured with a V-2000 photometer (CHEMetrics, Midland, VA, USA). Deionized water at room temperature was used for all the treatments. The temperatures of PAW bubbles generated by treatment A and B using ice cubes surrounding the treatment chamber were 9.8 ± 0.7 °C, and 12.3 ± 0.6 °C, respectively. The temperature of the PAW bubbles generated after treatment B without any ice surrounding the treatment chamber was 51.9 ± 1.2 °C.

### 4.4. EPR Spectroscopy

Electron spin resonance or Electron paramagnetic resonance (ESR/EPR) spectroscopy is an analytical technique that can detect unpaired electrons. In this study, the commonly used spin trap DMPO (5,5-dimethyl-1-pyrroline-N-oxide) (Dojindo Molecular Technologies, Inc., Rockville, MD, USA) was used to trap hydroxyl and superoxide radicals to be identified by EPR spectroscopy. Five milliliters of deionized water were mixed with 28 μL DMPO (8.97 M, undiluted and used without further purification) to make a 50 mM final concentration of DMPO. The solution was then treated with BSD ACP at different treatment times, using ice surrounding the treatment chamber. For free radical measurement, 50 μL of the sample was drawn into a 50 μL capillary tube (BRAND^®^ disposable BLAUBRAND^®^ micropipettes, Sigma Aldrich Canada Co., Item code: BR708733) and sealed at the end with Hemato-Seal™ Tube Sealing Compound (Item: 02–678, Fisherbrand^®^, Fisher Scientific Co., Ottawa, ON, USA). The microcapillary tube was then placed in a 5 mm thin wall quartz EPR tube (Wilmad Labglass, Vineland, NJ, USA) in an EPR resonator (Elexsys E-500 Spectrometer, Bruker Corp., Billerica, MA, USA) for analysis. The EPR spectroscopy settings: center field = 3504 G, sweep width = 80 G, modulation amplitude = 1 G, scans = 5, microwave frequency = 9.8 GHz, microwave power = 20 mW, receiver gain = 60 dB. The acquired EPR spectra were plotted with OriginLab 2022 (OriginLab Corporation, Northampton, MA, USA).

### 4.5. DON Quantification after PAW Bubble Treatments

First, 80 mL deionized water was added to 10 g of NIB grains in a beaker and treated directly by BSD ACP for 20 or 30 min to generate PAW bubbles. Ice cubes were placed around the treatment beaker to avoid temperature increase during the direct BSD ACP treatment (treatments A, B). In the indirect treatment (treatment C), 80 mL deionized water was treated with BSD ACP for 30 min and the generated PAW bubbles were used for steeping the NIB grains. To evaluate DON degradation during the malting process, the grains were steeped in PAW bubbles for 5 h, drained for 5 min, and air rested (19 h) at 15 ± 0.3 °C, 78 ± 3% RH after the treatments. The grains were dried at room temperature for 2 days and DON content was determined using Reveal^®^ Q+ test kits (Neogen, Lansing, MI, USA) based on the single-step lateral flow immunochromatographic assay. The limit of detection and the specificity of the kits for DON were 0.3 ppm, and 100%, respectively. The kits were validated previously by HPLC, and their accuracy confirmed to be >90%. For measuring DON content after direct CJ ACP treatment, 180 mL water was added to 10 g NIB grains inside the treatment chamber (Figure 4B), and the grains were treated for 30 min and 1 h to produce PAW bubbles followed by steeping (5 h overall), draining (5 min), and air rest (19 h) at 15 ± 0.3 °C, 77 ± 4% RH. The grains were then dried, and DON content was determined using Reveal^®^ Q+ test kits as above.

### 4.6. Grain Germination and Enzymatic Activity after PAW Bubble Treatments

Following a 2-day steeping in PAW bubbles and air rest, the NIB grains were allowed to germinate over a 3-day germination period inside a glass beaker. After/during BSD ACP, or CJ ACP treatments, the grains were steeped for 5 h in PAW bubbles followed by 5 min draining and 19 h air rest. The following day, the grains were treated using BSD ACP and CJ treatments again to produce PAW bubbles and steeped for 5 h, then 5 min draining, and 19 h air rest at 15 ± 0.3 °C and 77 ± 4% RH. Finally, the grains were washed with DW and drained, followed by the addition of 3 mL DW to 10 g NIB each day for 3 days during the germination period. The moisture content of the grains after 1st and 2nd day of steeping was determined as per the American Association for Clinical Chemistry (AACC) method by drying in an air oven for 2 h at 135 °C [53]. The α-amylase, β-amylase, and β-glucanase activities were determined in the green malt before germination using K-MALTA 07/20 malt amylase assay kit and S-ABG100 03/11 malt and bacterial β-glucanase assay kit (Megazyme, Bray, Wicklow, Ireland). Moreover, 5 g of germinated seeds (containing 110–130 seeds) were used to determine the percentage of germinated acrospires and rootlets separately. Grains with visible acrospires and rootlets with a length > 3 mm were considered to be germinated.

### 4.7. Analysis of Microbial Contamination and In Vitro Germination

Treated and untreated NIB grains were placed in Petri dishes (100 × 15 mm for PDA, 100 × 25 mm for WA) filled with potato dextrose agar (PDA) or water agar (WA) (Fisher Scientific, Montreal, QC, Canada) for mycological and germination analyses, respectively. Ten seeds were spread evenly in each Petri dish and three dishes were used for each replicate of the treatment. The seeds were not surface sterilized in order to evaluate the effect of ACP treatment on the naturally occurring microflora on the seeds. Fungal colony diameter and the proportion of seeds from which *F. graminearum* could be recovered was evaluated by incubating the seeds on PDA medium for 3 days at room temperature. Isolates of *F. graminearum* were identified based on their cultural and morphological characteristics [54]. Percentage germinated grains, shoot height, and number of *F. graminearum* colonies were recorded on the WA plates after 5 days’ incubation at room temperature.

### 4.8. DNA Extraction

For DNA extraction, 1–2 g of the treated and untreated NIB grains were ground to a powder in liquid nitrogen in a mortar with a pestle. Approximately 100 mg of the ground sample was used to extract total DNA using a DNeasy Plant Pro Kit (QIAGEN, Germantown, MD, USA). The quality of the extracted DNA was assessed with a NanoDrop1000 spectrophotometer (ThermoFisher Scientific) and diluted to a final concentration of 25 µg/mL.

### 4.9. Quantitative PCR

The fungal biomass of *F. graminearum* was estimated by qPCR analysis. Four *F. graminearum*-specific genes were targeted. The full sequence of the genes, including Trichothecene biosynthesis transcription regulator (*Tri6*, GenBank: AB017495.1), Terpene cyclase trichodiene synthase (*Tri5*; GenBank: KJ677974.1) [46], Elongation factor 1-alpha (*EF1 A*; GenBank: KX084040.1) [47], and Actin (NCBI Reference Sequence: XM_011328784.1) [48] were retrieved from the NCBI sequence database. Gene-specific primers (Table 6) were designed using Primer Express 3.0.1 (Applied Biosystems, Mississauga, ON, Canada). The primers were diluted to 20 µM in nuclease free water.

Quantitative PCR was performed on a QuantStudio 6 Flex system (Applied Biosystems, Canada). Each amplification reaction (10 µL volume) contained diluted DNA (2 µL), Fast SYBR Green Master Mix (5 µL; ThermoFisher Scientific) and 1 µL each of the forward and reverse primers in a Micro Amp Fast Optical well plate. Each reaction was performed in triplicate. A no template control, containing only nuclease-free water with master mix, was also included in the analysis. The qPCR was carried out using the following program: 95 °C for 20 s, followed by 40 cycles of 95 °C for 1 s and 60 °C 20 s. A melting curve (95 °C for 1 s, 60 °C for 20 s and 95 °C for 1 s) was generated to ensure the specificity of the amplification for each product. Relative quantification of *F. graminearum* genes was carried out using the 2^(−ΔΔCt)^ method [49] with *β-Actin* as internal reference gene and dry grain sample as a calibrator.

### 4.10. Statistical Analysis

SPSS (IBM SPSS v.27, Armonk, NY, USA) was used to determine significant differences (*p* < 0.05) by analysis of variance (ANOVA), followed by Duncan’s multiple range test. At least triplicate experiments were performed for all the experiments, and the data are expressed as the mean ± standard deviation.

## Figures and Tables

**Table 1:** Characteristics of PAW bubbles produced by different treatments used in this study. The characteristics of PAW were measured indirectly after ACP treatments, i.e., the generated PAW after plasma treatments was used for measuring the physicochemical properties of PAW.

	pH	ORP (mV)	H_2_O_2_ (ppm)	NO^−^_3_ (ppm)	NO^−^_2_ (ppm)	O_3_ (ppm)
DW	8.1 ± 0.2 ^a^	208 ± 33.2 ^c^	-	-	-	-
Treatment A ^1^	2.7 ± 0.06 ^d^	599 ± 1 ^a^	87.3 ± 11.9 ^b^	277.47 ± 19.6 ^b^	22.6 ± 2.2 ^b^	79.6 ± 8.2 ^b^
Treatment A + 5 h storage	2.64 ± 0.06 ^d^	594.3 ± 0.6 ^a^	54.7 ± 3.8 ^c^	299.6 ± 5.7 ^b^	16 ± 0.7 ^c^	34.1 ± 3.4 ^cd^
Treatment B without ice	2.5 ± 0.04 ^d^	606.7 ± 4.2 ^a^	15.6 ± 0.9 ^d^	238.6 ± 5.6 ^c^	6.3 ± 0.1 ^d^	22.7 ± 2.5 ^d^
Treatment B	2.5 ± 0.07 ^d^	608.7 ± 3.2 ^a^	133.5 ± 14.9 ^a^	465.2 ± 24.3 ^a^	33.7 ± 2.5 ^a^	103.8 ± 20 ^a^
Treatment B + 5 h storage	2.5 ± 0.04 ^d^	600 ± 1 ^a^	59 ± 6.2 ^c^	267.7 ± 40.1 ^bc^	16.9 ± 2.1 ^c^	42.8 ± 5.5 ^c^
Treatment D	5.99 ± 0.15 ^b^	236.3 ± 13.8 ^c^	0.3 ± 0.06 ^e^	4.24 ± 0.3 ^d^	0.057 ± 0.004 ^e^	0.08 ± 0.02 ^e^
Treatment D + 5 h storage	6.04 ± 0.14 ^b^	210.7 ± 10 ^c^	0.28 ± 0.06 ^e^	4.14 ± 0.4 ^d^	0.037 ± 0.006 ^e^	0.03 ± 0.006 ^e^
Treatment E	5.07 ± 0.2 ^c^	336 ± 31.4 ^b^	0.23 ± 0.04 ^e^	4.22 ± 0.69 ^d^	0.043 ± 0.003 ^e^	0.06 ± 0.01 ^e^

^1^ Treatment A: Direct 20 min BSD (surrounded by ice); Treatment B: Direct 30 min BSD (surrounded by ice); Treatment D: Direct 30 min CJ; Treatment E: Direct 1 h CJ. Temperature of PAW bubbles in treatments A, B without ice, B, and D + E were 9.8, 51.9, 12.3, and 22 °C, respectively. Values are expressed as the mean ± standard deviation. Values with different letters in the same column are significantly different (*p* < 0.05, *n* = 3).

**Table 2:** Effect of different PAW bubble treatments on DON reduction in NIB.

	DON Content (ppm)	Minimum (ppm)	Maximum (ppm)	Reduction (%)
Dry grain	5.2 ± 0.6 ^a^	4.6	5.9	0 ^b^
Control (5 h steeping) + 19 h air rest	3.3 ± 1 ^b^	2.6	4.4	36.9 ± 18.4 ^a^
Treatment A + 4 h 40 min steeping + 19 h air rest ^1^	3.3 ± 0.9 ^b^	2.4	4.1	37.6 ± 16.2 ^a^
Treatment B + 4.5 h steeping + 19 h air rest	2.2 ± 0.7 ^b^	1.5	3	57.3 ± 14.3 ^a^
Treatment C + 5 h steeping + 19 h air rest	3.6 ± 0.5 ^b^	3.2	4.1	31.2 ± 8.8 ^a^
Control D + 4.5 h steeping + 19 h air rest	3.3 ± 1.1 ^b^	2	4.9	37.7 ± 20.1 ^a^
Treatment D + 4.5 h steeping + 19 h air rest	2.9 ± 0.5 ^b^	2.6	3.5	44.6 ± 9.9 ^a^
Control E + 4.5 h steeping + 19 h air rest	3 ± 0.6 ^b^	2.5	3.7	43.3 ± 12.3 ^a^
Treatment E + 4 h steeping + 19 h air rest	3.1 ± 0.3 ^b^	2.9	3.5	40.1 ± 6.1 ^a^

^1^ Treatment A: Direct 20 min BSD (surrounded by ice); Treatment B: Direct 30 min BSD (surrounded by ice); Treatment C: Indirect 30 min BSD; Treatment D: Direct 30 min CJ; Treatment E: Direct 1 h CJ. Values are expressed as the mean ± standard deviation. Values with different letters in the same column are significantly different (*p* < 0.05, *n* ≥ 4).

**Table 3:** Effect of different PAW bubble treatments on germination of NIB.

	MC 1st Day Steeping (g Water/100 g Sample)	MC 2nd Day Steeping (g Water/100 g Sample)	Germinated Acrospire (%)	Germinated Rootlets (%)	α-Amylase (Units/g Dry Basis)	β-Amylase (Units/g Dry Basis)	β-Glucanase (Units/g Dry Basis)
Dry grain	-	-	-	-	12.6 ± 1.6 ^b^	31.6 ± 2.1 ^a,b^	22.3 ± 3.7 ^d^
DW + 5 h steeping + 19 h air rest	30.9 ± 0.6 ^a^	41.2 ± 0.8 ^a^	47.3 ± 7 ^a^	58.2 ± 2.7 ^a^	41.9 ± 0.7 ^a^	31.8 ± 1 ^ab^	33.3 ± 2.9 ^b,c^
Treatment A + 4 h 40 min steeping + 19 h air rest ^1^	31.7 ± 1.4 ^a^	42.7 ± 0.9 ^a^	15.1 ± 0.7 ^b^	70.1 ± 7 ^a^	45.1 ± 6.5 ^a^	33.4 ± 1 ^a^	39.9 ± 4.6 ^b^
Treatment B + 4 h 30 min steeping + 19 h air rest	32.9 ± 1.9 ^a^	42.6 ± 1.5 ^a^	3.6 ± 6.2 ^c^	18.5 ± 16.9 ^b^	39.5 ± 7.4 ^a^	30.9 ± 0.5 ^a,b^	26.7 ± 2.8 ^c,d^
Treatment C + 5 h steeping + 19 h air rest	31.5 ± 1.7 ^a^	41.7 ± 3 ^a^	38.1 ± 4.8 ^a^	75.08 ± 7.3 ^a^	-	-	-
Control D + 4 h 30 min steeping + 19 h air rest	33.36 ± 0.7 ^a^	42.5 ± 0.8 ^a^	41.1 ± 10.4 ^a^	63.7 ± 8.1 ^a^	-	-	-
Treatment D + 4 h 30 min steeping + 19 h air rest	33.46 ± 0.5 ^a^	43.6 ± 0.7 ^a^	44.1 ± 4.7 ^a^	68.5 ± 11 ^a^	39.2 ± 1.6 ^a^	30.2 ± 1.3 ^b^	51.6 ± 7.3 ^a^

^1^ Treatment A: Direct 20 min BSD (ice surrounding); Treatment B: Direct 30 min BSD (ice surrounding); Treatment C: Indirect 30 min BSD; Treatment D: Direct 30 min CJ. Values are expressed as the mean ± standard deviation. Values with different letters in the same column are significantly different (*p* < 0.05, *n* = 3).

**Table 4:** Effect of PAW bubble treatment on *F. graminearum* and grain germination ^1^.

	Average Colony Diameter on PDA (cm) ^2^	Number of *F. graminearum* Colonies on PDA	Number of *F. graminearum* Colonies on WA	Plant Shoot Length on WA (cm)	Germination % on WA
Dry grain	2.12 ± 0.06 ^a^	1.2 ± 0.8 ^a^	1.2 ± 1 ^a^	3.66 ± 0.06 ^b^	85.6 ± 1.9 ^a^
Control 5 h steeping + 19 h air rest	1.99 ± 0.07 ^b^	2.1 ± 1.4 ^a^	0.7 ± 0.5 ^a^	4.51 ± 0.69 ^a^	86.7 ± 3.3 ^a^
Treatment A + 4 h 40 min steeping + 19 h air rest	1.99 ± 0.08 ^b^	1.4 ± 1.4 ^a^	1.4 ± 1.1 ^a^	4.29 ± 0.36 ^ab^	87.8 ± 5.1 ^a^
Treatment C + 5 h steeping + 19 h air rest	1.89 ± 0.04 ^b^	2.3 ± 2.1 ^a^	1.4 ± 1.2 ^a^	4.38 ± 0.47 ^ab^	84.4 ± 5.1 ^a^
Treatment F + 4.5 h steeping + 19 h air rest	1.99 ± 0.05 ^b^	1.3 ± 0.9 ^a^	1.1 ± 1.1 ^a^	4.89 ± 0.34 ^a^	81.1 ± 1.9 ^a^

^1^ All the values in the table are the average number per Petri dish with 10 NIB seeds. ^2^: average colony diameter of all microflora, except *F. graminearum*, recovered from NIB seeds. Treatment A: Direct 20 min BSD (surrounded by ice); Treatment C: Indirect 30 min BSD; Treatment F: Direct 30 min BJ. PDA: potato dextrose agar; WA: water agar. Germination expressed as percentage of seeds that had shoot length more than 3 mm. Values are expressed as the mean ± standard deviation. Values with different letters in the same column are significantly different (*p* < 0.05, *n* = 3).

**Table 5:** Nomenclature of different treatments performed in this research.

Name	Treatment Name
Treatment A	Direct 20 min BSD (surrounded by ice)
Treatment B	Direct 30 min BSD (surrounded by ice)
Treatment C	Indirect 30 min BSD
Control D	Control 30 min CJ
Treatment D	Direct 30 min CJ
Control E	Control 1 h CJ
Treatment E	Direct 1 h CJ
Treatment F	Direct 30 min BJ

**Table 6:** The primers for *F. graminearum* genes.

	Forward PRIMER (5′–3′)	Tm (°C)	Reverse Primer	Product Size (bp)	Tm (°C)
Tri6	GCGGCATTACCGACAACACT	60	CGCACTGTTGGTTTGTGCTT	1247	58
EF1A	AAATTTTGCGGCTTTGTCGTA	58	GGCTTCCTATTGACAGGTGGTT	629	58
Actin	CGTCGCCCTTGACTTCGA	59	CCAAGGACAGAAGGCTGGAA	1544	59
Tri5	AGGAGCGCATCGAGAATTTG	59	TTGCCCAGCTGTATACAACCAT	1062	58

## Data Availability

Data is available on request.
